# Greenville Epsom Springs, Harrodsburgh, Ky.

**Published:** 1835

**Authors:** 


### VIII.—Greenville Epsom Springs, Harrodsburg, Ky.

In the second volume of this Journal we published an account of these springs. The great increase in the number of our subscribers since that time, will justify our republishing the results of the analysis of one of the fountains.

Sulphate of Magnesia, in large quantities—the characteristic ingredient.

Carbonate of Magnesia, in a small quantity.
Sulphate of Soda, do.
Sulphate of Lime, do.
Carbonate of Lime, a trace.
Iron, (probably a sulphate,) do.
Sulphuretted Hydrogen, do.

These springs have deservedly acquired much celebrity. The enterprizing proprietor, Dr. Graham, has expended on them thirty thousand dollars, and has comfortable accommodations for five hundred visitors.

